# Successful re-administration of Pazopanib in a patient with metastatic renal cell carcinoma and a history of Pazopanib-induced nephrotic syndrome: a case report

**DOI:** 10.1186/s12882-018-1181-1

**Published:** 2019-01-03

**Authors:** So-Yeon Jeon, Na-Ri Lee, Chang-Yeol Yim

**Affiliations:** 1 Department of Internal Medicine, Chonbuk National University Medical School, 20, Geonji-ro, Deokjin-gu, Jeonju, 54907 Republic of Korea; 2 Research Institute of Clinical Medicine of Chonbuk National University – Biomedical Research Institute of Chonbuk National University Hospital, Jeonju, Republic of Korea

**Keywords:** Pazopanib, Nephrotic syndrome, Metastatic renal cell carcinoma

## Abstract

**Background:**

Drug-induced nephrotic syndrome (NS) can be resolved by eliminating the causative agents. However, patients with metastatic cancer have not been previously reported to achieve complete recovery from anticancer drug-induced NS after discontinuation of treatment, because many patients die of cancer progression before NS is restored.

**Case presentation:**

A 67-year-old man presented with edema of both lower extremities. He received pazopanib therapy for recurrent metastatic renal cell carcinoma (mRCC) for 17 months. Laboratory examinations revealed 7484.58 mg/day of 24-h urine protein, 434 mg/dL of serum cholesterol, and 2.9 g/dL of serum albumin. He was diagnosed with NS, and pazopanib treatment was discontinued. Four months later, he completely recovered from NS. He was then treated with temsirolimus and nivolumab sequentially for > 26 months. Pazopanib was re-introduced following disease progression, and demonstrated antitumor effects for 7 months without NS recurrence.

**Conclusion:**

Pazopanib-induced NS can occur late in patients with mRCC, and its subsequent discontinuation can enable patients to completely recover from its adverse effects. Moreover, pazopanib treatment may be re-introduced without the recurrence of NS.

## Background

Pazopanib is a tyrosine kinase inhibitor targeting vascular endothelial growth factor (VEGF), platelet-derived growth factor (PDGF), and fibroblast growth factor (FGF) receptors, and c-Kit, among others [1]. Although a phase III study of pazopanib treatment for renal cell carcinoma (RCC) revealed no major adverse events in its safety profile, the incidence rate of proteinuria was approximately 10% (10–80%) [2, 3]. According to a Food and Drug Association (FDA) report, nephrotic syndrome (NS) occurs in patients who received pazopanib, especially in men aged ≥60 years, between 1 and 6 months after initiating the treatment. As of November 2017, DFA reported that 12,905 patients who received pazopanib exhibited side effects, 31 (0.24%) of whom developed NS [4]. NS can be reversed when the causative factor is eliminated, but discontinuation of treatment in cancer patients to enable recovery from NS may lead to disease progression and death. Herein, we present a case of a patient who received pazopanib for metastatic RCC (mRCC) and who subsequently developed NS; however, NS did not recur after complete recovery and subsequent re-introduction of pazopanib.

## Case report

A 67-year-old man complained of edema in both lower extremities. His medical history revealed that he had been diagnosed with stage I clear cell renal cell carcinoma (RCC) and underwent radical nephrectomy 8 years ago. Three years after the surgery, the cancer recurred and metastasized to the lungs and pancreas. The metastatic cancer was removed by performing pylorus-preserving pancreaticoduodenectomy and right upper lobectomy. The resected lung and pancreas indicated clear cell RCC. After metastasectomy, the cancer was classified as stage IV; he was administered with sunitinib (50 mg orally once daily), but 14 months later, the disease progressed. His medication was then changed from sunitinib to everolimus (10 mg orally once daily), but the disease continued to progress; therefore, pazopanib (800 mg orally once daily) was prescribed for mRCC for 17 months. When edema developed, immediate spot urine protein, albumin, and creatinine tests were performed. The albumin/creatinine (alb/cr) and protein/creatinine (prot/cr) ratios were 4300.64 mg/g and 5772.35 mg/g, respectively. A 24-h urine protein excretion test was performed and revealed 7484.58 mg/day of proteinuria, which was within the nephrotic range. Total cholesterol and serum albumin levels were 434 mg/dL and 2.9 g/dL, respectively. He was diagnosed with NS, and pazopanib treatment was discontinued. Other drugs to treat NS, such as angiotensin-converting enzyme inhibitor (ACEi) or glucocorticosteroids, were not administered, and only pazopanib treatment was discontinued. He had been taking calcium channel blocker (lacidipine 4 mg once daily) for hypertension since he had undergone nephrectomy, and his systolic blood pressure was less than 120 mmHg and diastolic blood pressure was less than 80 mmHg when he started taking pazopanib. But 2 months before the diagnosis of NS, systolic blood pressure increased to 140 mmHg, lacidipine was changed to amlodipine (5 mg twice daily), and blood pressure was regulated to normal range. The baseline serum creatinine levels were between 0.99 and 1.43 mg/dL after nephrectomy, with an average value of approximately 1.2 mg/dL. Serum creatinine level was 1.14 mg/dL when NS was diagnosed. In order to determine the cause of NS, a kidney biopsy should be performed. However, in consultation with a nephrologist, we decided not to undergo renal biopsy. Because the patient had a single kidney due to nephrectomy and grade 3 chronic kidney disease with estimated glomerular filtration rate between 50 and 60 ml/min/1.73m^2^, so there was a risk of aggravation renal failure after renal biopsy.

**Fig. 1:**
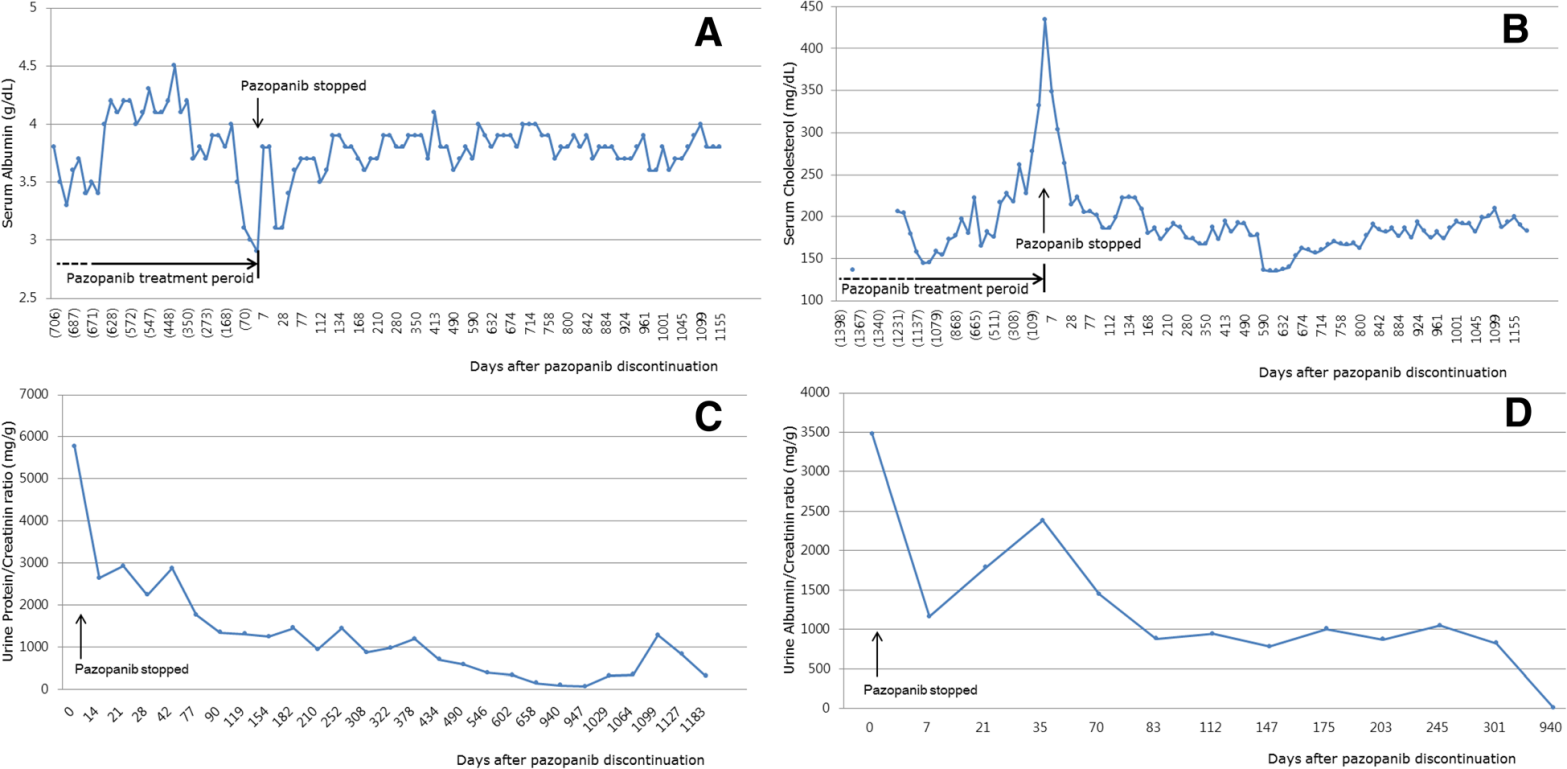
Changes in urinary protein, albumin, serum albumin and cholesterol after discontinuation of Pazopanib. Serum albumin(**a**) and cholesterol(**b**) levels before and after Pazopanib discontinuation. Changes in urine protein/creatinine ratio(**c**) and urine albumin/creatinine ratio(**d**) after discontinuation of Pazopanib

Pazopanib was discontinued for 3 months without further cancer treatment and changed to temsirolimus (25 mg intravenously, weekly) after disease progression, 1 month later. Proteinuria improved 3 months after pazopanib discontinuation, spot urine prot/cr ratio decreased to 1776.84 mg/g at 3 months and 948.31 mg/g at 7 months after discontinuation, and serum cholesterol levels normalized to 186 mg/dL at 4 months (Fig. 1). The patient had diabetes mellitus and exhibited trace proteinuria before pazopanib treatment; urine protein levels were completely restored to the previous levels. One month after discontinuation of pazopanib, blood pressure was lowered, and amlodipine was changed to lacidipine (4 mg once daily) again.

Temsirolimus was administered for 15 months, but as the disease progressed, nivolumab (100 mg intravenously, every other week) was administered for 11 months; however, the disease continued to progress. No anticancer agents were available that would be eligible for insurance coverage; thus, pazopanib was carefully re-administered for 7 months (800 mg/day as recommended dose), and the dose was not reduced. During this period, trace proteinuria was still present, and pazopanib was successfully administered without any NS recurrence. The spot urine prot/cr ratios upon resuming and discontinuing of pazopanib treatment were 69.05 mg/g and 318.88 mg/g, respectively. Serum creatinine levels were not significantly changed, and blood pressure was maintained in the normal range without any change in antihypertensive medication. Seven months after pazopanib retreatment, the disease progressed, and the patient participated in another clinical trial.

## Discussion

NS is a clinical syndrome characterized by proteinuria of ≥3.5 g per day, serum hypoalbuminemia of ≤3.0 g/dL, hyperlipidemia, and systemic edema. It is usually caused by diverticular glomerulonephritis such as primary glomerulonephritis, infections, medications, tumors, autoimmune diseases, diabetes mellitus, and preeclampsia. The mechanism of development is not well known.

Diagnosis should be made using single urine samples or 24-h urine collection. When using single urine samples, the albumin/creatinine ratio should be measured (≥30 mg is diagnostic). Kidney biopsies are usually needed to understand the cause and determine optimal treatment, but not necessary in patients with diabetes [5, 6]. In this case, we did not perform renal biopsy because the patient only had a single kidney due to previous radical nephrectomy and also had diabetes mellitus, which contributed to the concern on possible loss of renal function.

Pazopanib is a multitarget agent that best inhibits c-Kit and FGF, PDGF, and VEGF receptors. Inhibition of the VEGF signaling pathway is associated with proteinuria and hypertension. In addition to pazopanib, minimal change NS (MCNS) and focal and segmental glomerulosclerosis (FSGS) with bevacizumab, sorafenib, sunitinib, or axitinib treatment were also reported [7]. Although the mechanisms of development of these drug-induced MCNS/FSGS cases are not well understood, the drugs mediate the dysfunction of podocytes to form nephrotic-level proteinuria, and RelA, the master subunit of nuclear factor kappa-light-chain enhancer of activated B cells (NF-kB) and c-mip that induces podocyte disorder, is thought to play a key role here. VEGF-targeted agents increase RelA and alter the structure of podocyte actin cytoskeleton and induce proteinuria [8]. In addition to the mechanism associated podocyte, the other potential mechanisms of molecular targeted therapy induced proteinuria are as follows: subacute glomerular thrombotic microangiopathy, increased nitric oxide production, paraneoplastic glomerulopathy and so on [6]. There was no well-controlled randomized trial to treatment VEGF inhibitor induced NS, therefore only nonspecific treatment such as renin angiotensin system blockade are available.

The 5-year survival rate for mRCC is reportedly 8% [9]. Using the Heng score risk stratification, the 5-year survival rates were 41, 18, and 8% in the low-, intermediate-, and high-risk groups, respectively. The case reported herein was of a high-risk patient, although he remained alive for > 5 years after the recurrence. In a randomized double-blind, phase III study comparing pazopanib with a placebo, the median overall survival was 22.9 months when pazopanib was administered irrespective of previous treatment history and the median overall survival was 29.3 months according to other literature reports [2]. In our patient, the stable disease response to pazopanib was observed for 17 months. Pazopanib-induced NS mostly occurs between 1 and 6 months from treatment initiation, but our patient sustained for 17 months of use and relatively late-onset NS was observed. Xie et al. [10] previously demonstrated a 5.6 month (95% confidence interval [CI], 4.1–6.7 months) progression-free survival with second-line pazopanib; however, our patient presented a relatively long-term response to treatment with pazopanib. This is a rare but successful example with a poor prognosis, but with a long PFS. After discontinuation of pazopanib, NS was completely resolved, but the disease continued to progress. Although two other anticancer agents were administered sequentially, the disease progressed and pazopanib was re-administered for 7 months, with no NS recurrence noted. The mechanism of no NS recurrence is unclear and requires further research. But we think that this case may suggest that when the VEGF inhibitor is reused, it may show a different safety profile compared with that of the first use.

Cancer progression may be rapid, but mortality from NS itself may also increase. Adult patients with NS have been reported to exhibit a higher incidence of infection, renal dysfunction, acute renal failure, and hypertension in the future [5]. Considering this point, if cancer is accompanied with NS, acute death may more likely occur due to NS rather than the underlying malignancy. Pazopanib-induced kidney injury is reversible, but the survival is affected by the recovery of NS depends on the underlying tumor [11]. There has been no previous report of a patient with previously heavily treated metastatic cancer who discontinued anticancer treatment because of drug-induced NS and completely recovered from NS before death in mRCC. Moreover, successful administration of the same drug without NS recurrence has not yet been reported. By reporting this case, we suggest that patients with pazopanib-induced NS may be retreated with pazopanib, which may provide complete recovery from NS, especially if no other alternative chemotherapeutic treatment options are available.

## Conclusion

Pazopanib-induced NS in patients with mRCC may develop late in patients, and can be completely alleviated after discontinuation of treatment. If recovery was successful and in the absence of other treatment options for cancer, pazopanib can be re-administered with careful observation for potential NS recurrence.

## Abbreviations

ACEi: Angiotensin-converting enzyme inhibitor
FDA: Food and Drug Association
FGF: Fibroblast growth factor
FSGS: Focal and segmental glomerulosclerosis
MCSN: Minimal change NS
mRCC: Metastatic renal cell carcinoma
NS: Nephrotic syndrome
PDGF: Platelet-derived growth factor
RCC: Renal cell carcinoma
VEGF: Vascular endothelial growth factor
